# Variation of Blunt Traumatic Injury with Age in Older Adults: Statewide Analysis 2011–14

**DOI:** 10.5811/westjem.2016.9.31003

**Published:** 2016-10-07

**Authors:** Emily Earl-Royal, Frances Shofer, Dominique Ruggieri, Rosemary Frasso, Daniel Holena

**Affiliations:** * Perelman School of Medicine at the University of Pennsylvania, Department of Surgery, Philadelphia, Pennsylvania; † Perelman School of Medicine at the University of Pennsylvania, Department of Emergency Medicine, Philadelphia, Pennsylvania; ‡ University of Pennsylvania, Center for Public Health Initiatives, Perelman School of Medicine at the University of Pennsylvania, Philadelphia, Pennsylvania; § University of Pennsylvania, School of Nursing, Philadelphia, Pennsylvania; ¶ Perelman School of Medicine at the University of Pennsylvania, Center for Clinical Epidemiology, and Biostatistics, Philadelphia, Pennsylvania; || The Penn Injury Science Center at the University of Pennsylvania, Philadelphia, Pennsylvania; # Division of Traumatology, Surgical Critical Care and Emergency Surgery, Perelman School of Medicine at the University of Pennsylvania

## Abstract

**Introduction:**

Traumatic injury is a leading cause of death and disability in adults ≥ 65 years old, but there are few epidemiological studies addressing this issue. The aim of this study was to assess how characteristics of blunt traumatic injuries in adults ≥ 65 vary by age.

**Methods:**

Using data from the a single-state trauma registry, this retrospective cohort study examined injured patients ≥ 65 admitted to all Level I and Level II trauma centers in Pennsylvania between 2011 and 2014 (n=38,562). Patients were stratified by age into three subgroups (age 65–74; 75–84; ≥85). We compared demographics, injury, and system-level across groups.

**Results:**

We found significant increases in the proportion of female gender, (48.6% vs. 58.7% vs. 67.7%), white race (89.1% vs. 92.6% vs. 94.6%), and non-Hispanic ethnicity (97.5% vs. 98.6% vs. 99.4%) across advancing age across age groups, respectively. As age increased, the proportion of falls (69.9% vs. 82.1% vs. 90.3%), in-hospital mortality (4.6% vs. 6.2% vs. 6.8%), and proportion of patients arriving to the hospital via ambulance also increased (73.6% vs. 75.8% vs. 81.1%), while median injury severity plateaued (9.0% all groups) and the proportion of Level I trauma alerts (10.6% vs. 8.2% vs. 6.7%) decreased. We found no trend between age and patient transfer status. The five most common diagnoses were vertebral fracture, rib fracture, head contusion, open head wound, and intracranial hemorrhage, with vertebral fracture and head contusion increasing with age, and rib fracture decreasing with age.

**Conclusion:**

In a large cohort of older adults with trauma (n= 38,000), we found, with advancing age, a decrease in trauma alert level, despite an increase in mortality and a decrease in demographic diversity. This descriptive study provides a framework for future research on the relationship between age and blunt traumatic injury in older adults.

## INTRODUCTION

Older adults (≥65 years old) will comprise over 20% of the U.S. population by the year 2030; traumatic injury, including falls, is a leading cause of death and disability in this age group.^1^ Healthy People 2020, an initiative by the U.S. Department of Health and Human Services (HHS) that sets 10-year goals for disease prevention and health promotion, has declared it a national priority to reduce the rate of emergency department (ED) visits from falls in the elderly.^2^ However, despite the ubiquity of traumatic injury in older adults and its national recognition as a major public health issue, there is surprisingly little published about the epidemiology of blunt traumatic injury in this population.

While there are well documented differences in patterns of traumatic injury by age, the bulk of work on this topic has compared older adults as a collective to younger adults. 3 Little work has been done on the influence of gradations of age on blunt traumatic injury over 65 and on system-level factors that may influence these trends. Most of the literature on older adults and traumatic injuries is based on old data from the 1980s–1990s, from foreign countries like the United Arab Emirates, Australia, or Canada, focuses on prehospital triage criteria, or only evaluates hospital readmissions. 4–15

The aim of this study was to identify how characteristics of blunt traumatic injuries in adults ≥ 65, treated at an accredited trauma center in Pennsylvania (PA) between 2011 and 2014, vary by age. As the population of adults >65 continues to increase, accurate data on the rates and patterns of traumatic injury in this group are essential to improve understanding of the burden of blunt trauma in this vulnerable population. Such knowledge may increase our ability to prevent, screen, and treat trauma in older adults.

## METHODS

This study is a retrospective observational analysis of data collected from older adults hospitalized at trauma centers in PA from 2011–2014.

### Data

We obtained data from the Pennsylvania Trauma System Foundation (PTSF). Trauma centers are required to report utilization data to PTSF as a requisite for trauma center accreditation in PA; and as such, all trauma centers are strongly incentivized to accurately report information. Trained healthcare professionals entered the data in real time, and the data were abstracted and subject to review by trained PTSF auditors on a quarterly basis.

### Subjects

Patients were eligible if they were ≥ 65 years old and were admitted to an accredited trauma center for blunt traumatic injury in the state of PA between January 1, 2011, and December 31, 2014. Blunt injuries are defined as injuries from a blunt object or from collision with a blunt surface, such as falls and motor vehicle collisions (MVCs).

In the absence of established age strata in the ≥65 year old groups, we established our own cut-offs: 65–74, 75–84, and ≥85. These groups divided the data into roughly three equal parts, allowing similar levels of power to facilitate inter-group comparisons.

### Variables

We compared demographic, injury, and system-level variables across the age groups. Demographic variables included age, sex, race, ethnicity, insurance type, and pre-existing conditions (PECs). Injury variables included mechanism of injury (MOI) (as determined by external injury code, or e-code), diagnosis (as determined by ICD-9 code), place of injury, injury severity score (ISS), death in-hospital, and discharge destination. System variables included mode of transport to hospital, transfer status (which included both transfer into and out of a hospital), and trauma alert level (I, II, III, or trauma consult, with I being the highest level of alert possible and consult being the lowest level of alert possible at a given facility).

### Statistical Analysis

To compare demographic variables, injury characteristics, and system variable across age groups, we performed bivariate analysis using Kruskal-Wallis for continuous variables and chi-squared or Cochran-Armitage test of trend for categorical variables where appropriate. All analysis was done using STATA software version 14.0. We considered a two-tailed alpha value of less than .05 to be statistically significant. We did not adjust for multiple comparisons.

This study was deemed exempt by the institutional review board, as this was publicly available, de-identified data.

## RESULTS

Of the 38,562 admissions meeting criteria, 28.8% were 65–74 years old; 36.6% were 75–84 years old; and 34.6% were≥85 years old (Table 1). We found significant increases in the proportion of female gender, (48.6% vs. 58.7% vs. 67.7%), white race (89.1% vs. 92.6% vs. 94.6%), and non-Hispanic ethnicity (97.5% vs. 98.6% vs. 99.4%) across advancing age across age groups, respectively. Ten PECs had a frequency of ≥10%. From most to least frequent, these were hypertension (HTN), coronary artery disease (CAD), psychiatric disease, thyroid disease, arthritis, reversible anticoagulant therapy, dementia, antiplatelet therapy, congestive heart failure (CHF), and cerebrovascular disease (CVD). All of these PEC significantly increased with age (p<0.001), except for psychiatric disease, which decreased with age (p<0.001).

Falls were the most common mechanism of injury and increased with age (69.9% vs. 82.1% vs. 90.3%, p<0.001, Table 2). MVCs, the second most common mechanism of injury, decreased with age (p<0.001). As age increased, median injury severity stayed the same (9.0) while the 75^th^ percentile decreased (14.0 vs. 13.0 vs. 12.0), but in-hospital mortality increased (4.6% vs. 6.2% vs. 6.8%). In all age groups, the majority of injuries took place at home. The five most common diagnoses in descending order were fracture of the vertebral column; fracture of the rib(s), sternum, larynx, and trachea; intracranial hemorrhage (ICH); open wound of the head; and facial contusion. The proportion of vertebral column fractures and facial contusions increased with age (p<0.001), while the proportion of rib and surrounding structures fractures decreased with age (p<0.001). No trend was observed between age and ICH (p=0.564) or age and open head wound (p=0.306).Most patients, regardless of age, were brought in via ambulance or fire rescue (Table 3), and this increased significantly with age. Conversely, patients arriving via private vehicle or walk-in decreased (p<0.001). No trend was observed between age and the proportion of patients transferred (p=0.283). Trauma alert level decreased with increased age (p<0.001).

## DISCUSSION

Our study examined how demographic, injury, and system-level characteristics of blunt traumatic injuries vary by age in adults ≥ 65 years old treated at a trauma center in PA from 2011–2014 and found that trauma alert levels trend downward with age, while in-hospital mortality trends upward.

### Demographics

The vast majority of older adults (95%) receiving care at a trauma institution in PA were white and non-Hispanic; females were also overrepresented (67%), especially as age increased. These characteristics increased with age. According to the U.S. Census, Pennsylvania’s total population is 83% white and 78% non-Hispanic, so it appears that minority groups were underrepresented in the injury data.^16^ However, an examination of PA census data at a more granular level revealed the racial, ethnic, and gender proportions of the current study accurately reflect the demographics of PA, in that ~95% of PA residents ≥85 are white and non-Hispanic.^17^ Therefore, it may be that the racial discrepancies seen in the current study were less likely due to racial or ethnic discrepancy in trauma center use and more likely a reflection of the skewed distribution of racial demographics of older adults, a trend that may reflect larger societal issues related to health status and longevity.

Several of the most common PECsare known intrinsic risk factors for fall; CAD, CVA, and arthritis have been shown to increase the risk of falling.^18^ While some PEC increase risk, other PEC, such as thyroid disease and reversible anticoagulant therapy, may worsen damage after a trauma and contribute to increased ISS. For example, chronic hyperthyroidism has been shown to increase risk of fracture, while levothyroxine, a common medication for thyroid disease, has also been implicated in increasing fracture risk.^19^ Reversible anticoagulant therapy, which many older adults take for thrombosis prophylaxis and treatment, exacerbates the effects of a fall by leading to persistent bleeding. Further analysis is needed to determine whether certain PEC are associated with higher mortality or ISS for the patients in this dataset. If an association is found, these findings may suggest that the medical and public health communities could benefit from a universal screening program for those on anticoagulants or a reevaluation of the risk-benefit ratio of such therapy.

### Injury patterns

**Figure 1:**
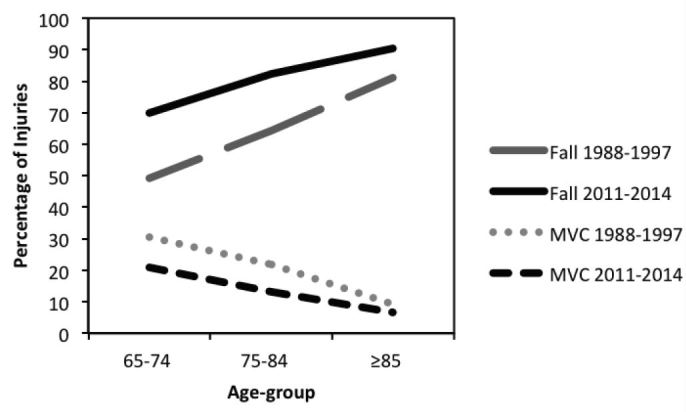
Change over time in injury mechanism from Richmond et al. study (using the same Pennsylvania trauma data source from 1988–1997) to current study (2011–2014). *MVC,* motor vehicle collision.

**Figure 2:**
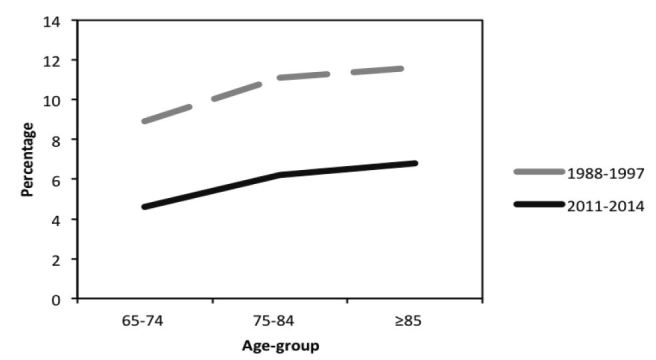
Change over time in in-hospital mortality rate from Richmond et al. study (using the same Pennsylvania trauma data source from 1988–1997) to current study (2011–2014). *MVC*, motor vehicle collision.

While the injury patterns seen here generally match broad trends seen elsewhere, there are several differences worth noting. Compared to earlier data from a similar study by Richmond et al. that used the same Pennsylvania Trauma Outcome Survey (PTOS) data source from 1988–1997, the proportion of older adults being injured by falls has grown dramatically: 49.2% vs. 69.9% for age 65–74; 64.2% vs. 82.2% for age 75–84; and 81.1% vs. 90.3% for ≥85, (Figure 1).^6^ Similarly, the proportion of MVCs has decreased dramatically: 30.4% vs. 21.0% for age 65–74; 21.7% vs. 13.2% for age 75–84; and 9.2% vs. 6.5% for ≥85 (Figure 1). In-hospital mortality rates have also decreased from >10% in Richmond’s study to <6% in the current study, though the general trend of increased mortality with increased age persists (Figure 2). 6 Richmond’s study did not report median ISS, but the mean ISS for that cohort was 11.7, which is higher than the mean ~7 or median 9.0 ISS seen in the current study. One possible explanation for the increase in falls and simultaneous decrease in MVCs, injury severity, and in-hospital mortality might be successful public health campaigns that have instituted car safety measures, such as airbags and increased seat belt use, both of which could contribute to the decrease in ISS and in-hospital mortality. While one could make the point that older adults may drive less than younger adults, which could influence injury patterns, we would assert that the MVC injuries noted here reflect both MVC drivers and passengers.

### System patterns

We observed no relationship between age and the proportion of patients transferred in or out. However, all age groups in this study had transfer rates approximately three times that seen in injured adults ≥70 in another recent study.^10^ It is unclear why our data would have a transfer rate three times that of data for a similar study. One possible explanation might be that the Ichwan study looked at traumatic injuries taken via ambulance to all hospital types, not just to trauma centers, while the current study looked only at injuries taken to a trauma center. As such, the average ISS in our study may have been higher than that of the Ichwan study.

Trauma alert level trended down as age increased (p<0.001). At first glance, this decrease in trauma level may seem appropriate, given that the ISS IQR also decreased with age. However, previous work has suggested that older adults with traumatic injuries may be systematically under-triaged, regardless of their injury severity.^12^, ^20^–^23^ Another possible explanation for the increased mortality among older adults, including those with lower injury severity, has suggested that older adults may require a unique set of triage criteria due to their unique physiologic reserve.^20^, ^23^–^26^

Our study has many strengths. For example, it uses a large, statewide data sample that is mandatory, collected in real time, and regularly audited by trained professionals. These strengths help assure the study is adequately powered, avoids recall bias, and has a low rate of erroneous information.

## LIMITATIONS

This study has several limitations. The data for this study only include one state, PA, and therefore our findings may not be generalizable to other populations. Furthermore, since PTOS only records information on patients admitted to a trauma center, we were unable to compare patients to those treated at a non-trauma center. Finally, to protect patient privacy the PTOS database does not allow for patient linkage. As such, it is possible that some patients may appear in the database multiple times or that the same injury may appear more than once if a patient was transferred between trauma centers. We attempted to minimize these effects by reporting transfer data and by excluding non-blunt injuries, such as burns, which we surmised might have a high rate of transfer to specialty centers and therefore a high rate of repeat entry in the database.

## CONCLUSION

Our study found that, for older adult trauma patients, trauma alert levels trend downward with age, but in-hospital mortality trends upward. When compared to earlier studies that used the same dataset, it is clear that mechanisms of injury are changing: falls now cause the vast majority of injuries in older adults seen at trauma centers while MVCs are responsible for a decreasing percentage of injuries in this population. This study identified multiple areas upon which to focus injury prevention and public health research for older adults, including triage appropriateness, impact of pre-existing conditions, and possible barriers to trauma center care.

## Figures and Tables

**Table 1:** Demographic characteristics (including co-morbidities) by age group (all comparisons listed are significant with p <0.001) in study of blunt traumatic injury in adults over 65.

	65–74		75–84		≥85	
			
Age group	N	(%)	n	(%)	n	(%)
Number	11,0888	(28.8)	14,115	(36.6)	13,359	(34.6)
Female	5,390	(48.6)	8,279	(58.7)	9,042	(67.7)
Race						
White	9,876	(89.1)	13,068	(92.6)	12,635	(94.6)
Black	718	(6.5)	601	(4.3)	417	(3.1)
Asian	113	(1.0)	125	(0.9)	82	(0.6)
Other/unknown	381	(3.4)	321	(2.2)	225	(1.7)
Ethnicity – Hispanic	274	(2.5)	199	(1.4)	84	(0.6)
Pre-existing conditions*						
Hypertension	7,433	(67.1)	10,607	(75.2)	10,599	(79.3)
Coronary artery disease	2,608	(23.5)	4,456	(31.6)	4,414	(33.0)
Psychiatric disease	2,778	(25.1)	3,133	(22.2)	3,003	(22.5)
Thyroid disease	1,828	(16.5)	3,080	(21.8)	3,453	(25.9)
Arthritis	1,593	(14.4)	2,538	(18.0)	2,741	(20.5)
Reversible anticoagulant	1,227	(11.1)	2,692	(19.1)	2,338	(17.5)
Dementia	489	(4.4)	2,087	(14.8)	3,577	(26.8)
Antiplatelet therapy	1,245	(11.2)	1,905	(13.5)	1,796	(13.4)
Congestive heart failure	910	(8.2)	1,698	(12.0)	2,224	(16.7)
Cerebral vascular disease	979	(8.8)	1,719	(12.2)	1,664	(12.5)

* Pre-existing conditions present in >10% of the sample are included in this table

**Table 2:** Injury characteristics by age group.

	65–74		75–84		≥85	
			
Age group	n	(%)	n	(%)	N	(%)
Injury mechanism						
Fall	7,749	(69.9)	11,589	(82.1)	12,058	(90.3)
MVC	2,332	(21.0)	1,864	(13.2)	872	(6.5)
Place of injury						
Home	5,887	(53.1)	8,538	(60.5)	8,004	(59.9)
Street/highway	2,752	(24.8)	2,206	(15.6)	1,069	(8.0)
Public building	736	(6.6)	976	(6.9)	623	(4.7)
Residential institution	440	(4.0)	1,402	(9.9)	3,113	(23.3)
Other/unspecified	1,273	(11.5)	993	(7.0)	550	(4.1)
ISS median (IQR)	9.0	(5.0–14.0)	9.0	(5.0–13.0)	9.0	(5.0–12.0)
Diagnosis**						
Fracture of vertebral column without mention of spinal cord injury	2,497	(22.5)	3,209	(22.7)	3,298	(24.7)
Fracture of rib(s), sternum, larynx, and trachea	2,795	(25.2)	2,948	(20.9)	2,646	(19.8)
Intracranial hemorrhage*	1,316	(11.9)	1,888	(13.4)	1,586	(11.9)
Open wound of head (excluding eye)*	2,172	(19.6)	3,176	(22.5)	3,063	(22.9)
Contusion of face, scalp, and neck except eye(s)	2,167	(19.5)	2,746	(19.4)	2,690	(20.1)
Died in hospital	509	(4.6)	877	(6.2)	902	(6.8)

* Not statistically significant

** Diagnoses present in > 10% of the sample were included in this table.

*MVC,* motor vehicle collision; *ISS,* injury severity score; *IQR,* interquartile range.

**Table 3:** System characteristics by age group (trauma alert Level I is the highest possible alert level, alert Level II is the second highest, and alert Level III and trauma consult are the lowest possible alert levels at a given facility).

	65–74		75–84		≥85	
			
Age group	n	(%)	n	(%)	n	(%)
Mode of transport						
Ambulance or fire rescue	8,160	(73.6)	10,698	(75.8)	10,830	(81.1)
Private vehicle or walk-in	2,262	(20.4)	2,727	(19.3)	1,937	(14.5)
Other/unknown	666	(6.0)	690	(4.9)	592	(4.4)
Transfer (in or out)*	3,617	(32.6)	4,676	(33.1)	4,288	(32.1)
Trauma alert called						
I	1,177	(10.6)	1,154	(8.2)	898	(6.7)
II	2,956	(26.7)	3,311	(23.5)	2,842	(21.3)
III or trauma consult	4,163	(37.6)	6,051	(42.9)	6,353	(47.6)

* Not statistically significant
